# Enhanced Right-Chamber Remodeling in Endurance Ultra-Trail Athletes Compared to Marathon Runners Detected by Standard and Speckle-Tracking Echocardiography

**DOI:** 10.3389/fphys.2017.00527

**Published:** 2017-07-25

**Authors:** Kristian Ujka, Luca Bastiani, Gennaro D'Angelo, Bruna Catuzzo, Alessandro Tonacci, Simona Mrakic-Sposta, Alessandra Vezzoli, Guido Giardini, Lorenza Pratali

**Affiliations:** ^1^Insitute of Clinical Physiology, National Research Council Pisa, Italy; ^2^Mountain Medicine Center, Ospedale Regionale Umberto Parini Aosta, Italy; ^3^Institute of Bioimaging and Molecular Physiology, National Research Council Milan, Italy

**Keywords:** extreme physiology, endurance sports, cardiac remodeling, speckle tracking echocardiography, cardiovascular diseases

## Abstract

**Background:** Strenuous and endurance exercise training have been associated with morphological and functional heart remodeling. Two-dimensional speckle-tracking echocardiography (STE) is a novel technique that allows an accurate quantification of global myocardium deformation. Our aim was to evaluate together left and right cardiac remodeling in different long-distance running athletes: marathon runners (42 km) (M) and endurance mountain runners (>300 Km) (UT).

**Methods:** A total of 92 athletes (70 males, 76%) including 47 M [age 45 ± 7 years; training: 18 (9–53) years^*^days/week], 45 UT [age 42 ± 9, training: 30 (15–66) years^*^days/week] underwent conventional echocardiography and STE (Beyond Diogenes 2.0, AMID) during the agonistic season.

**Results:** Right ventricle (RV) end-diastolic area (*p* = 0.026), fractional area changing (FAC) (*p* = 0.008) and RV global longitudinal strain (GLS) were significantly increasedin UT athletes. Furthermore, UT showed larger right atrium (RA) volume (*p* = 0.03), reduced RA GLS and significantly increased RA global circumferential strain (GCS) compared to M. After adjustment for age, sex, and HR as covariates, UT showed a reduced RA GLS (OR 0.907; CI 0.856–0.961) and increased RV FAC (OR 1.172; CI: 1.044–1.317) compared to M.

**Conclusion:** Athletes enrolled in UT endurance activities showed RV and RA morphological and functional remodeling to increased preload in comparison with M runners characterized by increased RV FAC and reduced RA GLS. Follow-up studies are needed to better assess the long-term clinical impact of these modifications. 2D STE is a useful tool for investigating the deformation dynamic in different sports specialties.

## Introduction

“Athlete's heart” is now a widely acknowledged term indicating a specific phenotype of cardiac morphologic remodeling to long-term physical activity (Fagard, 2003). However, the remodeling may be different among different sports according to the type of hemodynamic (volume and/or pressure) overload (Mitchell et al., 2005). Many studies using two-dimensional (2D) echocardiography have improved our understanding of “athlete's heart.” Endurance exercise is a dynamic (aerobic) exercise mainly characterized by volume overload, which induces specific remodeling characterized by left ventricle (LV) and left atrium (LA) dilation and an increased relative wall thickness (RWT) and LV mass without any systolic or diastolic dysfunction (Pelliccia et al., 1991, 2005; Pluim et al., 2000). On the other hand, right ventricle (RV) remodeling has been poorly studied in athletes, mainly due to the complex anatomy and location of the RV, which makes it difficult to study by conventional echocardiography, and to the wide heterogeneity of its function (Jurcut et al., 2010). Some studies have found RV and right atrium (RA) enlargement in elite athletes (Henriksen et al., 1996; Erol and Karakelleoglu, 2002). While in recent years participation in endurance sports has increased, several concerns have been raised about the harmful effects these sports may have on cardiac morphology and function and the risk of cardiac arrhythmias (Calvo et al., 2012; La Gerche et al., 2015).

2-D speckle-tracking echocardiography (STE) is a novel, non-invasive echocardiographic technique that allows an accurate quantification of global myocardium deformation along the three-dimensional (3D) geometrical axis (Teske et al., 2007; Mondillo et al., 2011). STE is a valuable tool for pathophysiological assessment, but still has very limited clinical applications even in patients with cardiovascular disease. Recent studies using STE have improved our understanding of the functional adaption of “athlete's heart.” Although there is disagreement as to whether athletes have higher LV strain compared to controls, many studies agree that decreased LV strain in athletes is an early sign of LV dysfunction typically found in patients with hypertensive or hypertrophic cardiomyopathy (Richand et al., 2007; Cappelli et al., 2010; D'Ascenzi et al., 2016). Few authors have studied RV and RA strain in athletes but results are controversial. While some authors showed that RV longitudinal strain was greater in athletes compared to controls (Pagourelias et al., 2013; Esposito et al., 2014), others found reduced RV longitudinal strain in athletes (Teske et al., 2009b). Regarding RA, 2D STE studies showed RA remodeling in elite athletes characterized by increased RA volume, reduced RA strain, and better diastolic function compared to controls (D'Ascenzi et al., 2013; Pagourelias et al., 2013). Controversial results may be due to the lack of standardization among the different STE software algorithms and to the fact that athlete's heart remodeling depends on the type and intensity of training (D'Ascenzi et al., 2016).

The aim of this study was to assess the morphological and functional remodeling of athlete's heart in different long-term intensive endurance athletes (ultra-trail endurance runners and marathon runners), using standard 2D echocardiography and STE.

## Methods

### Study population

Our study population was made of 45 ultra-endurance athletes specialized in ultra-trail running (UT) (>300 km) and 47 marathon runners (42 km) (M) recruited using local advertising. Inclusion criteria were: age 18–65, previous participation in competitive sports of their category, apparent good health status and written informed consent. Exclusion criteria were: known cardiovascular or pulmonary disease or symptoms and absence of informed consent.

The study protocol was approved by the institutional Ethics Committee of the Aosta Valley Hospital (n.895; 31/8/2015) and followed the guidelines of the Helsinki Declaration. All volunteers were informed regarding the design and purposes of the study and gave written informed consent.

Subjects were studied at rest in a quiet, temperature-controlled room. Medical history was collected with particular attention to the assessment of traditional cardiovascular risk factors. Brachial blood pressure (BP) and heart rate (HR) were measured at rest with subject in supine position using an automatic BP monitoring system, while oxygen saturation (SpO_2_) was measured using a portable pulsoximeter (Pulse-oximeter Model Tuff-Sat, Datex-Ohmeda, General Electric Healthcare Clinical System, Helsinki, Finland). Three measurements were taken within a 3-min interval and averaged. Body weight, body mass index (BMI), and total body water were measured using the bioelectrical impedance analysis (TanitaSC-331S Body Composition Analyzer; Tanita Inc., Arlington Heights, IL, USA). Training time, expressed as years of training^*^days of training per week was also assessed and expressed as double product (Training Time = years of training^*^days/week) while training intensity was estimated as km run per week (km/week).

### Standard 2D echocardiography

Standard 2D echocardiography was performed using a portable echo machine with a 2.5–3.5 MHz cardiac probe (Vivid I, General Electric Healthcare Clinical System). Right and left ventricle function was assessed according to American Society of Echocardiography (ASE) and European Association of Cardiovascular Imaging (EACVI) guidelines for chamber quantification (Lang et al., 2015). Interventricular septum and posterior wall thickness and LV end-diastolic diameter were measured in parasternal long-axis view and RWT and LV mass index (LVMI) were calculated according to guidelines (Lang et al., 2015). LV end-systolic and end-diastolic volumes were measured in the apical four-chamber view and ejection fraction (EF) was calculated by the modified biplane Simpson's method (Lang et al., 2015). Cardiac Output (CO) was measured multiplying LV outflow tract time-velocity integral, measured using pulse wave Doppler, by its cross-sectional area and heart rate. Right ventricle basal and middle diameters were measured in apical four-chamber view to assess any right ventricle dilation typically found in athletes. RV systolic function was assessed using tricuspid annular plane systolic excursion (TAPSE) measured with caliper in M-mode echocardiography according to ASE+EACVI guidelines for RV function (Rudski et al., 2010).

LV and RV diastolic function were assessed in four-chamber view using pulsed Doppler. Mitral and tricuspid early (E) and atrial (A) velocities were measured using pulsed Doppler and mitral and tricuspid E/A ratio was calculated. Tissue Doppler imaging was measured from the four-chamber view using pulsed-wave Doppler for both mitral and tricuspid annulus. Early (e') and atrial (a') diastolic velocities were measured at the lateral and septal borders of the mitral annulus and at the tricuspid lateral annulus. The ratio between mitral and tricuspid E velocity and e' (E/e') and e'/a' ratio was then calculated (Nagueh et al., 2009; Rudski et al., 2010). Systolic pulmonary artery pressure (sPAP) was estimated from the peak velocity of the tricuspid regurgitation jet by continuous flow Doppler and the systolic RA pressure estimated from the inferior vena cava diameter and its respiratory excursion (0–15 mmHg) using the formula: sPAP = 4V^2^ + RA pressure (Yock and Popp, 1984).

All 2D echocardiography parameters obtained from the UT and M were compared to normal values according the current recommendations of ASE+EACVI (Lang et al., 2015).

### 2-D speckle-tracking echocardiography

STE was performed from an apical four-chamber view using a narrow-sector gray scale images for all four chambers, with temporal resolution of 60–90 frames/s. Gain, compression, and dynamic range were optimized to enhance myocardial definition with standardized depth, frequency, and insonation angle for all athletes. 2D strain analysis was performed offline by the same expert sonographer (G.D.), not blinded to the group allocation, using semi-automatic strain software (Beyond Diogenes 2.0, AMID). After a region of interest was manually traced along the endocardial border in end-systole and end-diastole, the software automatically calculated the chamber's volume and global strain along the three axes (longitudinal, radial, and circumferential). LV strain was measured in the apical in the four-chamber view. LV end-systolic and end-diastolic volume and global longitudinal (GLS), radial (GRS), and circumferential (GCS) strain were automatically calculated from the software (Supplementary Figure 1). LA and RA endocardial border was manually traced in four-chamber view and the end-systolic volume and strain along the three axes were calculated. End-systolic (ESA), end-diastolic area (EDA), fractional area changing (FAC), and global RV strain were also measured from an apical four-chamber image focused on the right ventricle.

### Statistical analysis

Statistical analysis was performed using SPSS software version 21.0 for Windows (IBM Corp., Armonk, NY, USA). Continuous variables were expressed as mean ± standard deviation for normally distributed variables and in median and percentiles for non-normally distributed variables, while categorical data were expressed in percentages. *T*-test for independent samples was used to assess differences between means for normally distributed variables while Mann-Whitney Test was used for non-normally distributed variables. Normal distribution was tested using Kolmogorov-Smirnov test. Categorical variables were analyzed using χ^2^ test and Fisher's exact test when appropriate. Multiple regression models were made to adjust strain parameters with standard echocardiographic parameters. With the aim of evaluating strain outcomes between the different groups studied (M and UT), a binary logistic regression model with backward step-wise elimination was performed. We defined as dependent variable the group UT (1 = UT; 0 = M). Results are reported as odds ratio (OR) with a 95% confidence interval (adjusted for sex, age, and heart rate). Intra-observer reproducibility was assessed using the intraclass correlation coefficient (ICC). The Posteriori Power Analysis was based on the difference of means of several cardiac indexes (between groups). The Power Analysis for RV GCS with 45 subjects for UT Group and 47 subjects for M Group was 0.82. For RV GLS with 45 subjects for UT Group and 47 subjects for M Group, respectively, the estimated power for a two-sample means test was above 0.60 (0.61).

## Results

Demographic and clinical characteristics of the study population are shown in Table 1. The groups were comparable for age, sex, and BMI and body surface area. No significant difference in HR or BP was found between the UT and M. The prevalence of diabetes, dyslipidemia and obesity was low and with no difference between groups. Training intensity (Km/week) was not significantly different, while training time (day/week^*^year) was significantly higher in UT.

**Table 1:** Demographic and clinical characteristics of the study population.

	**UT (*n* = 45)**	**M (*n* = 47)**	*P*-value^*^
Age (years)	42 ± 9	45 ± 8	0.15^*^
Men (%)	84.4	65.3	0.57^§^
SBP (mmHg)	133 ± 13	130 ± 19	0.32^*^
DBP (mmHg)	77 ± 10	77 ± 10	0.83^*^
Heart Rate (bpm)	55.6 ± 6.9	54.1 ± 7.6	0.86^*^
BSA (m^2^)	1.84 ± 0.16	1.84 ± 0.21	0.88^*^
BMI (kg/m^2^)	22.7 ± 2.4	22.8 ± 2.3	0.79^*^
TI (km/week)	66.5 ± 39.1	51.8 ± 31.2	0.078^#^
TT (days/week^*^year)	30 (15–66)	18 (9–53)	**0.03**^#^
Hypertension (%)	4.4	6.1	1.00^§^
Diabetes (%)	0	2	1.00^§^
Smoke (%)	0	10.2	0.06^§^
Dyslipidemia (%)	0	6.1	0.24^§^
Obesity (%)	0	0	α

*SBP, Systolic Blood Pressure; DBP, Diastolic Blood Pressure; BSA, Body surface area; BMI, Body Mass Index; TI, Training Intensity; TT, Training Time. α: χ^2^ test was not performed because the variable was constant*.

* Parametric test (Student's t-test);

# Non-parametric test (Mann-Whitney test);

§ *χ^2^ test*.

*Significant p-values (p < 0.05) are marked in bold*.

### 2-D Doppler echocardiography

**Figure 1:**
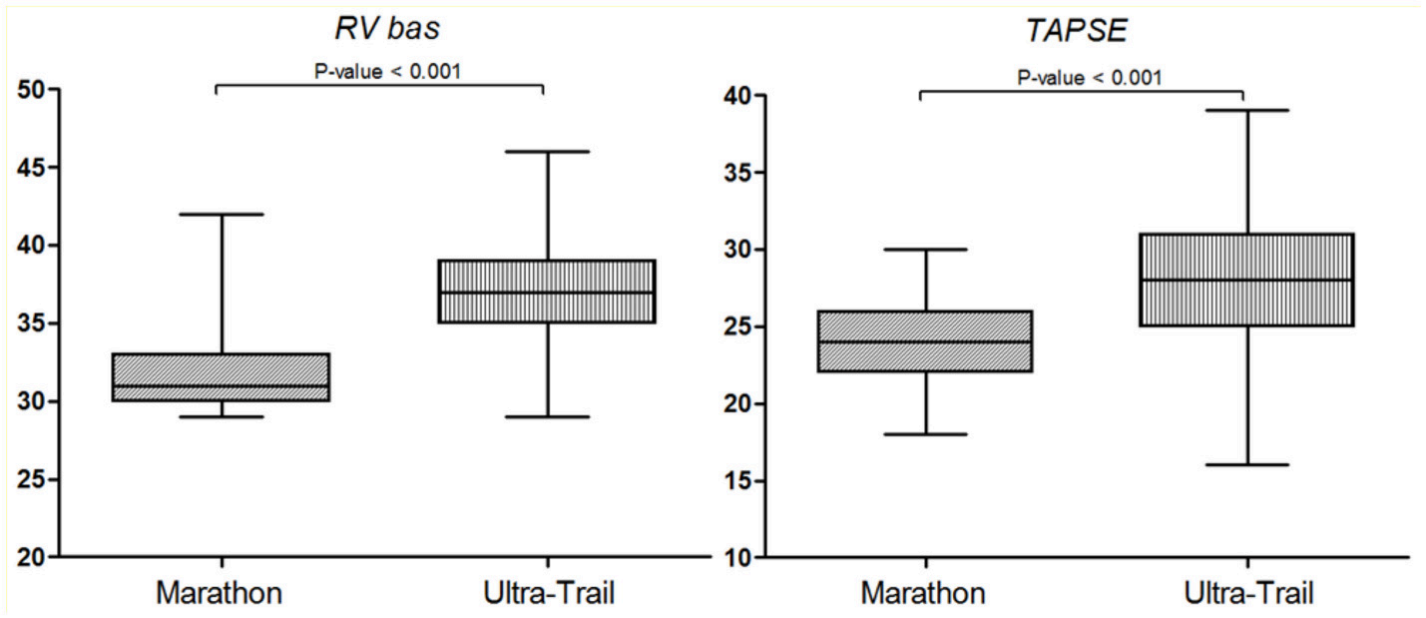
Dot plots showing the difference between groups for RV baseline diameter and TAPSE. RV bas, Right ventricle baseline diameter; TAPSE, Tricuspid annular plane systolic excursion.

Standard echocardiography was successfully performed in all subjects (100% feasibility). The main 2D and Doppler parameters are shown in Table 2. In Supplementary Table 1 the 2D Doppler echocardiogram parameters were compared to normal values according to American Society of Echocardiography and European association of Cardiovascular Imaging (Lang et al., 2015). The study of LV diastolic function showed a higher mitral E/A ratio and mean e' velocity and a lower of E/e' ratio in UT runners compared to M that can be linked to a reduced LV filling pressure. However, both groups presented normal mitral inflow pattern and normal diastolic function (Supplementary Table 1). RV diameters, RV FAC and TAPSE were significantly increased in UT (Figure 1). RV diastolic function was normal in both groups (Supplementary Table 1). However, tricuspid E/A and E/e' ratio were significantly increased in UT. Moreover, UT athletes showed an increased systolic and diastolic PAP and an increased inferior vena cava diameter compared to M runners. No significant difference in LV systolic function or LV dimension was found between groups.

**Table 2:** Two-dimensional echocardiography and Doppler parameters.

	**UT (*n* = 45)**	**M (*n* = 47)**	*P*-value^*^
RWT	0.37 ± 0.0	0.37 ± 0.04	0.71
LVMI	88.6 ± 17.7	87.5 ± 11.6	0.94
LVEDV (ml)	108 ± 28	114 ± 24	0.25
EF (%)	61.6 ± 6.5	62.6 ± 2.2	0.33
CO (l/min)	4.4 ± 1.3	4.2 ± 1.3	0.52
RV bas (mm)	36.7 ± 3.6	32.1 ± 2.7	<**0.001**
RV mid (mm)	31.9 ± 5.1	27.1 ± 2.0	<**0.001**
RV FAC (%)	43.3 ± 12.5	36.4 ± 6.5	**0.002**
TAPSE	28.0 ± 0.8	24.0 ± 0.4	<**0.001**
E	78 ± 15	78 ± 13	0.82
DT	224 ± 47	238 ± 32	0.10
E/A mitral	1.6 ± 0.5	1.4 ± 0.3	**0.04**
e' mean mitral	12.7 ± 3.2	11.1 ± 1.9	**0.003**
E/e' mitral	5.6 ± 2.9	7.2 ± 1.3	**0.001**
E/A tric	1.8 ± 0.1	1.3 ± 0.3	<**0.001**
E/e' tric	5.5 ± 0.4	4.5 ± 0.2	**0.008**
sPAP (mmHg)	28.6 ± 5.5	24.3 ± 3.7	<**0.001**
mPAP (mmHg)	19.2 ± 3.3	16.6 ± 2.2	<**0.001**
PVR	2.8 ± 1.4	2.4 ± 1.2	0.14
TPR	23.8 ± 7.7	24.6 ± 7.5	0.62
IVC (mm)	19.3 ± 5.4	14.2 ± 2.4	<**0.001**

LVEDD, Left Ventricle End Diastolic Volume; EF, Ejection Fraction; CO, Cardiac Output; RV bas, right ventricle basal diameter; RV mid, Right Ventricle middle diameter; TAPSE, Tricuspid Annulus Plane Systolic Excursion; sPAP, Systolic Pulmonary Artery Pressure; mPAP, Mean Pulmonary Artery Pressure; PVR, Pulmonary Vascular Resistances; TPR, Total Peripheral Resistances; IVC, Inferior Vena Cava;

* *Parametric test (Student's t-test). Significant p-values (p < 0.05) are marked in bold*.

### 2-D speckle-tracking echocardiography

**Figure 2:**
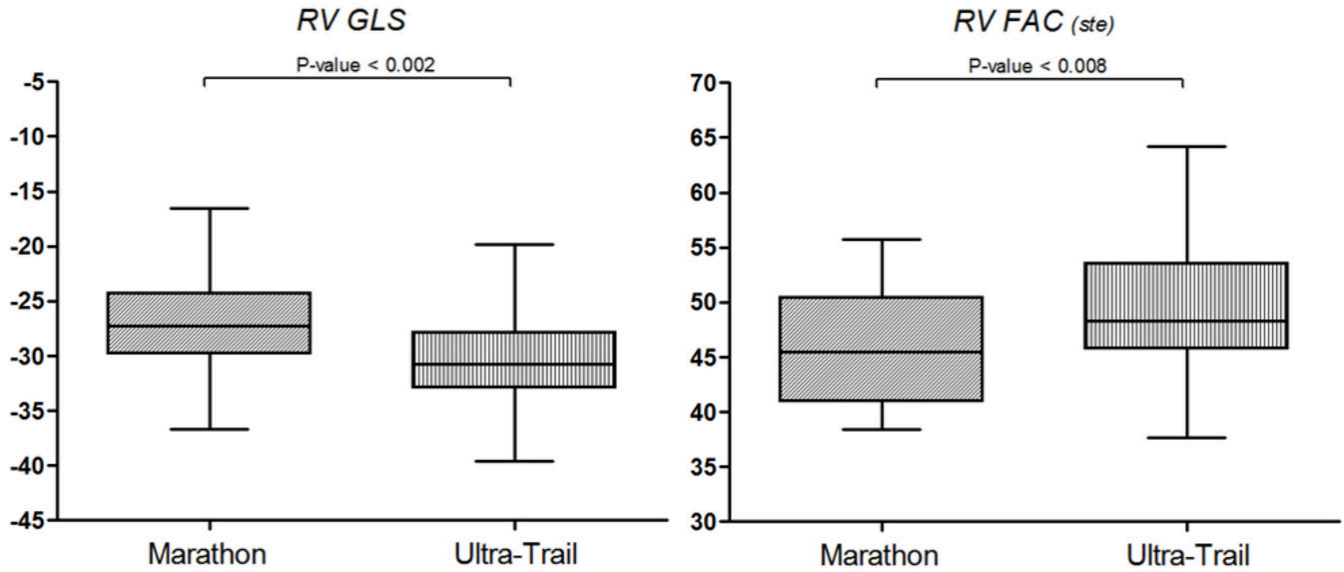
Dot plots showing the difference between groups for RV GLS and RV FAC. RV GLS, Right ventricle global longitudinal strain; RV FAC (ste), Right ventricle fractional area changing.

**Figure 3:**
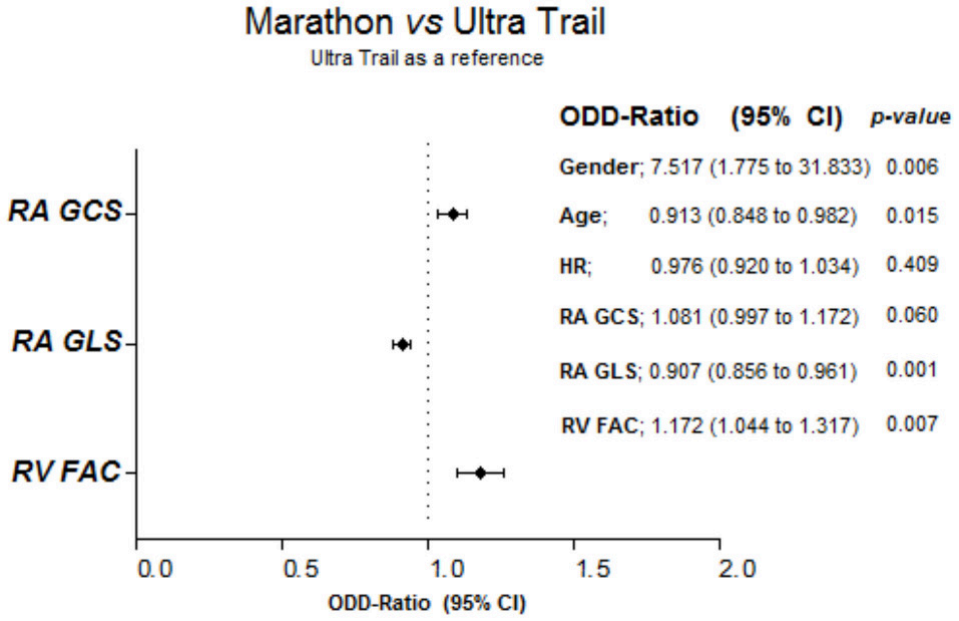
Bimodal logistic regression models. RA, Right Atrium; RV, Right Ventricle; GLS, Global Longitudinal Strain; GCS, Global Circumferential Strain; FAC, Fraction Area Changing; HR, Heart Rate. The non-significant covariates from backward elimination were progressively removed and only the variables shown in the figure remained significant.

STE of the four-chamber view was feasible in 88 out of 92 subjects (95%). Four subjects (two UT and two M) were excluded due to low acoustic window or to inappropriate image acquisition. The main STE parameters are shown in Table 3. RV EDA_(ste)_ and RV FAC_(ste)_ (Figure 2) were significantly increased in UT compared to M, similarly to the 2D echocardiography results. Moreover, RV GLS was significantly increased in UT (Figure 2). UT athletes were also characterized by increased RA volume, reduced RA GLS and an increased RA GCS compared to M runners. Inferior vena cava diameter was found to be an independent predictor of RA GLS (β = 0.204, *p* = 0.04). Binary logistic regression models were made using a backward step-wise method as shown in Figure 3. After being adjusted for age, sex, and HR as covariates, UT showed a reduced RA GLS (OR 0.907; CI 0.856–0.961) and increased RV FAC (OR 1.172; CI: 1.044–1.317) compared to M.

**Table 3:** Two-dimensional speckle tracking derived parameters of the study population.

	**UT (*n* = 43)**	**M (*n* = 45)**	*P*-value^*^
LV EDV_(ste)_	126.3 ± 20.7	117.6 ± 25.8	0.08
LV EF_(ste)_	62.4 ± 3.5	63.3 ± 3.2	0.21
LV GLS_(ste)_	−27.6 ± 4.2	−28.6 ± 3.4	0.25
LV GCS	−28.7 ± 4.8	−29.3 ± 4.6	0.49
LV GRS	66.6 ± 11.2	69.8 ± 8.9	0.13
RV EDA_(ste)_	21.2 ± 4.6	18.8 ± 4.9	**0.026**
RV FAC_(ste)_	49.2 ± 5.9	45.9 ± 5.0	**0.008**
RV GLS	−30.4 ± 4.4	−27.3 ± 4.5	**0.002**
LA ESV_(ste)_	59.7 ± 15.7	59.6 ± 18	1.00
LA GLS	35.0 ± 12.2	36.5 ± 11.1	0.55
LA GCS	26.4 ± 12.5	28.7 ± 11.3	0.39
LA GRS	−32.5 ± 8.5	−34.4 ± 6.9	0.27
RA ESV_(ste)_	63.9 ± 23.2	53.8 ± 17.2	**0.03**
RA GLS	31.6 ± 9.6	37.1 ± 13.5	**0.03**
RA GCS	22.2 ± 8.9	17.5 ± 6.4	**0.004**
RA GRS	−30.3 ± 2.3	−31.2 ± 7.4	0.52

LV, Left ventricle; RV, Right Ventricle; LA, Left atrium; RA, Right Atrium; EDV, End Diastolic Volume; ESV, End Systolic Volume; EF, Ejection Fraction; GLS, Global Longitudinal Strain; GC, Global Circumferential Strain; GRS, Global Radial Strain; FAC, Fraction Area Changing;

* *Parametric test (Student's t-test). Significant p-values (p < 0.05) are marked in bold*.

Intra-operator reproducibility for STE was tested for ten subjects, reading the same images as shown in Supplementary Table 2 (Supplementary Material). ICC ranged from 0.714 to 0.990, *p* < 0.05.

## Discussion

In this study we evaluated the morphological and functional characteristics of cardiac remodeling in different long-term intensive endurance athletes, UT and M using standard echocardiography and STE. UT showed no significant differences regarding LV and LA volumes or STE measurements. Regarding the right chambers, UT runners showed RV and RA remodeling compared to M runners characterized by increased RV diameters and RA volume in presence of an “enhanced” RV function as indicated by increased RV FAC, TAPSE, RV GLS, RA GCS, and reduced RA GLS. The RV dilation could represent a RV adaption to bradycardia and to the increased venous return, which is associated with increased systolic function, as reported in previous studies (Erol and Karakelleoglu, 2002; D'Andrea et al., 2013; Major et al., 2015).

Moreover, the RV diastolic function, assessed as tricuspid E/A ratio and E/e' ratio, was normal. We also found increased sPAP and mPAP in UT compared to M. The increased PAP is very frequent in athletes and has also been considered a consequence of a physiological adaption to increased venous return and not to LV dysfunction (D'Andrea et al., 2011). In fact, LV E/e', an estimation of LV filling pressure, was not increased in UT while the inferior vena cava was significantly dilated suggesting remodeling due to chronic endurance exercise.

With STE we observed an increased RV FAC_(ste)_ and RV GLS in UT athletes compared to M, confirming the “enhanced” RV systolic function evaluated by 2D echocardiography in UT elite athletes. Moreover, being an UT athlete was found to be an independent significant predictor of having increased RV FAC even after adjusting for age, sex, HR, and other strain covariates compared to M. On the other hand, RV GLS was not significantly different between UT and M when adjustment was made for other covariates. These findings suggest an increased RV function in UT athletes, which may represent training-induced remodeling to long-lasting volume overload. In fact, although training intensity was not significantly different between groups, UT showed increased training time.

From strain analysis of RA we found an increased RA volume, a reduced RA GLS and an increased RA GCS among UT athletes. However, when adjustment was made for age, sex, HR, and strain covariates, RA volume and RA GCS were not significantly different, while RA GLS remained independently reduced in UT, suggesting decreased RA systolic contraction in this group of athletes.

In accordance with current literature (D'Andrea et al., 2015), we did not find any significant difference in LV mass, volume or function between athletes. STE-derived parameters were comparable and Tissue Doppler-derived markers of diastolic function were normal in all groups. However, UT athletes showed increased E/A ratio and reduced E/e', indicating a better diastolic function. Furthermore, no significant difference in LA dimensions of function was found between groups.

### Physiological hypothesis of “enhanced” RV function in UT

During acute exercise the muscles start to contract, creating compression on the veins and thus increasing the venous flow. The increased venous return to the right heart dilates the RV and RA, and according to the Starling mechanism increases the stroke volume which together with the exercise-induced tachycardia increases the cardiac output able to maintain an adequate perfusion to the muscle (Opie, 1998). Trained athletes, chronically enrolled in dynamic physical activity, undergo many hemodynamic modifications of the heart characterized by increased volume load with a constant or minimal increase of pressure load which induces a LV remodeling characterized by an increased LV mass without any significant increase in wall thickness, known as physiological hypertrophy. The LV hypertrophy is associated with a reduced HR due to parasympathetic tone predominance and an improved diastolic function. However, the remodeling due to the volume load may be even greater on the RV and RA, which has a thinner wall thickness compared to the LV (Opie, 1998; Fagard, 2003). The increased preload together with the longer diastolic filling time due to the bradycardia induce RV and RA dilation. However, despite the resting bradycardia, the athlete's heart is able to maintain cardiac output by increasing the stroke volume, which can explain the present results of RVs enlarged and with increased systolic function in UT athletes compared to M. On the other hand, the resting bradycardia increases the diastole's total duration and in association with higher preload, modifies the diastolic pattern: increasing E mitral wave (early phase of LV diastolic filling), reducing A mitral wave (atrial contribution), and thus increasing the E/A ratio, as we found in UT athletes. Furthermore, the reduced contribution of the atrial systole at rest (A mitral wave) may represent a pattern of atrial remodeling rather than an atrial dysfunction (Opie, 1998; D'Ascenzi et al., 2013). In fact, endurance athletes showed a lower atrial contribution at rest compared to the exercise where the atrial systole contribution to ventricular filling may increase, as a reserve mechanism to afford the increased pre-load.

### Comparison with previous studies

To understand the real significance of heart remodeling in athletes it is fundamental to differentiate physiological adaptation from inherited cardiomyopathy such as arrhythmogenic right ventricle cardiomyopathy or hypertrophic cardiomyopathy. Recently, many STE studies have focused their attention on assessing RV remodeling in endurance athletes compared to active subjects, showing contradictory results.

Teske and coauthors showed a reduced RV global and regional strain in elite athletes (training intensity 24.2 ± 5.7 h/week) and athletes (training intensity 12.5 ± 2.3 h/week) in comparison with controls, this reduction being more pronounced in athletes with marked RV dilation (Teske et al., 2009b). La Gerche et al. showed that rest RV function was reduced in 40 endurance athletes compared to non-athletes as assessed by 2D and STE, while during exercise the RV strain and strain rate increased progressively with HR without any significant difference between groups, showing no difference in RV contractile reserve (La Gerche et al., 2012). Probably, in presence of RV dysfunction in resting condition in healthy athletes, a further evaluation of RV function during exercise should be employed. Very recently, Bohm et al. studied 33 endurance athletes (including 16 elite athletes) compared with 33 healthy controls. The subjects were studied by cardiopulmonary exercise test, 2D, TDI and Strain echocardiography to assess LV and RV morphology and function, and by contrast enhanced cardiac magnetic resonance. In the final results, although the athletes showed LV and RV enlargement in presence of normal biventricular function, a pathological late enhancement was detected in only one athlete, confirming how LV and RV dilation represents physiological remodeling and is not associated with ventricular dysfunction or risk of arrhythmias (Bohm et al., 2016). The contradictory results of published studies are principally due to the fact that neither the type of athletes (kind of sports, training history, period of evaluation) studied nor the methods used were homogenous, in particular for the differences in strain software analysis. STE can be an useful tool for distinguishing between the RV physiological remodeling found in athletes and arrhythmogenic RV dysplasia (D'Ascenzi et al., 2016), but reference values for LV and RV measured should be published for healthy subjects and for athletes, a wider clinical use (D'Ascenzi et al., 2016). As matter of fact, while both of these conditions may be characterized by the RV dilation (Bauce et al., 2010), patients with arrhythmogenic RV dysplasia have been shown to have reduced RV strain compared to controls (Teske et al., 2009a).

Regarding RA, studies on atrial function in athletes are still very limited. In a recent study, D'Ascenzi et al. studied 100 athletes and 78 controls using STE found an increased RA volume and a reduced RA peak longitudinal strain in athletes compared to controls (D'Ascenzi et al., 2013). More recently, Pagourelias et al. also found increased RA volume and reduced RA early diastolic strain rate without any significant differences in RA function showing RA remodeling to volume afterload (Pagourelias et al., 2013). Our finding are in concordance with these studies regarding RA volume. In this study we found a reduced RA GLS in UT athletes compared to M in absence of any RV diastolic dysfunction, which seem to represent physiological atrial remodeling to the increased volume load.

The literature on LV strain in athletes is also controversial. Simsek et al. found increased values of GLS in marathon athletes compared to controls (Simsek et al., 2013), while Capelli et al. found no difference in GLS between endurance athletes and controls (Cappelli et al., 2010; Caselli et al., 2015). More recently, Caselli et al. studied 200 Olympic athletes enrolled in different disciplines (including both isometric and endurance sport activities); although being normal, LV GLS was lower compared to controls, with no difference related to the sport discipline (Caselli et al., 2015). Similar results were also found from Richard et al. in professional soccer players (Richand et al., 2007). However, when compared to patients with hypertrophic cardiomyopathy, athletes had increased GLS LV strain (Richand et al., 2007; Cappelli et al., 2010; Butz et al., 2011). Thus, although there is no agreement on whether athletes have increased LV strain compared to controls, authors agree that a reduction in GLS should be considered an early marker of LV systolic dysfunction (D'Ascenzi et al., 2016).

The modifications of LA volume in athletes have been previously reported as a component of “athlete's heart” (Pelliccia et al., 2005), while its function has been neglected. In a recent study, D'Ascenzi et al. showed that LA modification in athletes goes beyond LA enlargement. In soccer players, the author found an increased mitral E/A ratio and reduced LA peak contraction strain which correlated with early diastolic annular velocity (D'Ascenzi et al., 2011). In this study, although we did not find any significant difference in LA strain between UT and M athletes, UT showed increased E/A ratio, increased early diastolic annular velocity and reduced E/e' ratio, indicating a small yet better diastolic function compared to M, confirming what was found by D'Ascenzi.

### Limitations

Some limitations for this study must be stated. First, for technical reasons, strain analysis of the LV, and LA was performed only in the apical four-chamber view. Another limitation may be that the sonographer was not blinded to the group allocation which may represent an investigator bias. Finally it must be stated that our study was conducted during the agonistic season when the athletes were undergoing intense training. It would be important to assess whether the modification we found is a transitory response to the training program or a permanent remodeling phenotype of the athletes. Follow-up studies may be needed for a better understanding of the clinical impact of these results.

## Conclusions

Athletes enrolled in UT endurance activities showed RV and RA morphological remodeling associated with an enhanced function in comparison with M runners, which may represent a training-induced remodeling to long-lasting volume overload. Follow-up studies are needed to better assess the long-term clinical impact of these modifications. 2D STE is a useful tool for investigating the deformation dynamic in different sport specialties.

## Author contributions

KU was involved in the clinical examinations, data analysis and interpretation, writing the manuscript, and final approval of the manuscript submitted. LB was involved in the statistical analysis, final writing, and final approval of the paper. GD was involved in the data analysis and final approval of the paper. BC was involved in clinical examination and final approval of the manuscript submitted. AT was involved in the final approval of the manuscript. SM was involved in the study design, clinical examinations, and final approval of the manuscript. AV was involved in the clinical examinations and final approval of the paper. GG was involved in the study design, and final approval of the manuscript submitted. LP was involved in the study design, data analysis, and interpretation, in the drafting and final writing and approval of the paper.

### Conflict of interest statement

The authors declare that the research was conducted in the absence of any commercial or financial relationships that could be construed as a potential conflict of interest.

## Abbreviations

2D: Two-Dimension
LV: Left Ventricle
LA: Left Atrium
RWT: Relative Wall Thickness
RV: Right Ventricle
RA: Right Atrium
STE: Speckle-Tracking Echocardiography
3D: Three-Dimensional
UT: Ultra-Trail
M: Marathon
BP: Blood Pressure
HR: Heart Rate
SpO_2_: Oxygen Saturation
BMI: Body Mass Index
ASE: American Society of Echocardiography
EAE: European Association of Echocardiography
LVMI: LV Mass Index
EF: Ejection Fraction
CO: Cardiac Output
TAPSE: Tricuspid Annular Plane Systolic Excursion
E: Early mitral/tricuspid velocity
A: Atrial mitral/tricuspid velocity
e': Early diastolic velocity
a': Atrial diastolic velocity
sPAP: Systolic Pulmonary Artery Pressure
GLS: Global Longitudinal Strain
GCS: Global Circumferential Strain
GRS: Global Radial Strain
EDA: End Diastolic Area
ESA: End Systolic Area
FAC: Fraction Area Changing
OR: Odds Ratio
ICC: Intraclass Correlation Coefficient.
