# Supplementary material for: Enhanced Right-Chamber Remodeling in Endurance Ultra-Trail Athletes Compared to Marathon Runners Detected by Standard and Speckle-Tracking Echocardiography
Source: Front Physiol. 2017 Jul 25;8:527. doi: 10.3389/fphys.2017.00527 (PMC5524917; doi:10.3389/fphys.2017.00527)
Supplement: Supplementary file 1 [file Table1.DOCX]

# Supplementary Table 1

|  | UT | M | Reference^1^ |
| --- | --- | --- | --- |
| E/A Mitral (mean, sd) | 1,6 (0,5) | 1,4 (0,3) | 1,53 (0,40) |
| P-value* | ns | < 0,001 |  |
| e' mean Mitral | 12,7 (3,2) | 11,1 (1,9) | 17,6 (2,8) |
| P-value* | < 0,001 | < 0,001 |  |
| E/e' Mitral | 5,6 (2,9) | 7,2 (1,3) | < 8 |
| P-value* | ns | ns |  |
| LV EDV (mL) (mean, sd) | 88,6 (17,7) | 87,5 (11,6) | 106 (22) |
| P-value* | < 0,001 | < 0,001 |  |
| LV EF (biplane) (mean, sd) | 61,6 (65) | 62,6 (22) | 62 (5) |
| P-value* | ns | ns |  |
| LVMI (mean, sd) | 88,6 (17,7) | 87,5 (11,6) | < 115 |
| P-value* | ns | ns |  |
| RWT (mean, sd) | 0,37 (0,0) | 0,37 (0,04) | < 0.42 |
| P-value* | ns | ns |  |
| RV basal diameter (mm) (mean, sd) | 36,7 (3,6) | 32,1 (2,7) | 33 (4) |
| P-value* | < 0,001 | < 0,05 |  |
| RV middle diameter (mm) (mean, sd) | 31,9 (5,1) | 27,1 (2,0) | 27 (4) |
| P-value* | < 0,001 | ns |  |
| TAPSE (mm) (mean, sd) | 28 (0,8) | 24 (0,4) | 23 (3,5) |
| P-value* | < 0,05 | ns |  |
| RV FAC (%) (mean, sd) | 43,3(12,5) | 36,4 (6,5) | 49 (7) |
| P-value* | < 0,05 | < 0,001 |  |
| IVC (mean, sd) | 19,3 (5,4) | 14,2 (2,4) | < 25 |
| P-value* | ns | ns |  |
| E/A tric (mean, sd) | 1,8 (0,1) | 1,3 (0,3) | 1.4 |
| P-value* | < 0,001 | < 0,05 |  |
| E/e' tric (mean, sd) | 5,5 (0,4) | 4,5 (0,2) | <6 |
| P-value* | ns | ns |  |
| sPAP (mean, sd) | 28,6 (5,5) | 24,3 (3,7) | < 35 |
| P-value* | Ns | ns |  |

**Supplementary table 1.** **Comparison of 2D ehocardiographic parameters between ultra-trail, marathon athletes and reference values for healthy subjects.** LV EDV: Left Ventricle End Diastolic Volume; EF: Ejection Fraction; LVMI: Left Ventricle Mass Index; RWT: Relative Wall Thickness; TAPSE: Tricuspid Annulus Plane Systolic Excursion; IVC: Inferior Vena Cava; sPAP: Systolic Pulmonary Artery Pressure; *****: one sample t-test. ^1^ *Guidelines for chamber quantifications 2015*
