# Supplementary material for: Enhanced Right-Chamber Remodeling in Endurance Ultra-Trail Athletes Compared to Marathon Runners Detected by Standard and Speckle-Tracking Echocardiography
Source: Front Physiol. 2017 Jul 25;8:527. doi: 10.3389/fphys.2017.00527 (PMC5524917; doi:10.3389/fphys.2017.00527)
Supplement: Supplementary file 2 [file Table2.DOCX]

# Supplementary Table 2

|  | **Intra-operator ICC** | **P value** |
| --- | --- | --- |
| **LV EDV** _(ste)_ | 0.981 | <0.001 |
| **LV GLS** | 0.766 | 0.021 |
| **LV GRS** | 0.814 | 0.010 |
| **LV GCS** | 0.742 | 0.028 |
| **RV EDA** _(ste)_ | 0.990 | <0.001 |
| **RV GLS** | 0.714 | 0.038 |
| **RV FAC** | 0.917 | 0.001 |
| **LA ESV** _(ste)_ | 0.966 | <0.001 |
| **LA GLS** | 0.770 | 0.020 |
| **RA ESV** _(ste)_ | 0.984 | <0.001 |
| **RA GLS** | 0.782 | 0.023 |

**Supplementary table 2**. Intra-operator reproducibility of the same Speckle Tracking Echocardiography (ste) images using Intra-class correlation coefficient (ICC). LV: Left ventricle; RV: Right Ventricle; LA: Left atrium: RA: Right Atrium; EDV: End Diastolic Volume; FAC: Fraction Area Changing; ESV: End Systolic Volume; GLS: Global Longitudinal Strain; GCS: Global Circumferential Strain; GRS: Global Radial Strain;
