# Supplementary material for: Enhanced Right-Chamber Remodeling in Endurance Ultra-Trail Athletes Compared to Marathon Runners Detected by Standard and Speckle-Tracking Echocardiography
Source: Front Physiol. 2017 Jul 25;8:527. doi: 10.3389/fphys.2017.00527 (PMC5524917; doi:10.3389/fphys.2017.00527)
Supplement: Supplementary file 3 [file Image1.PDF]

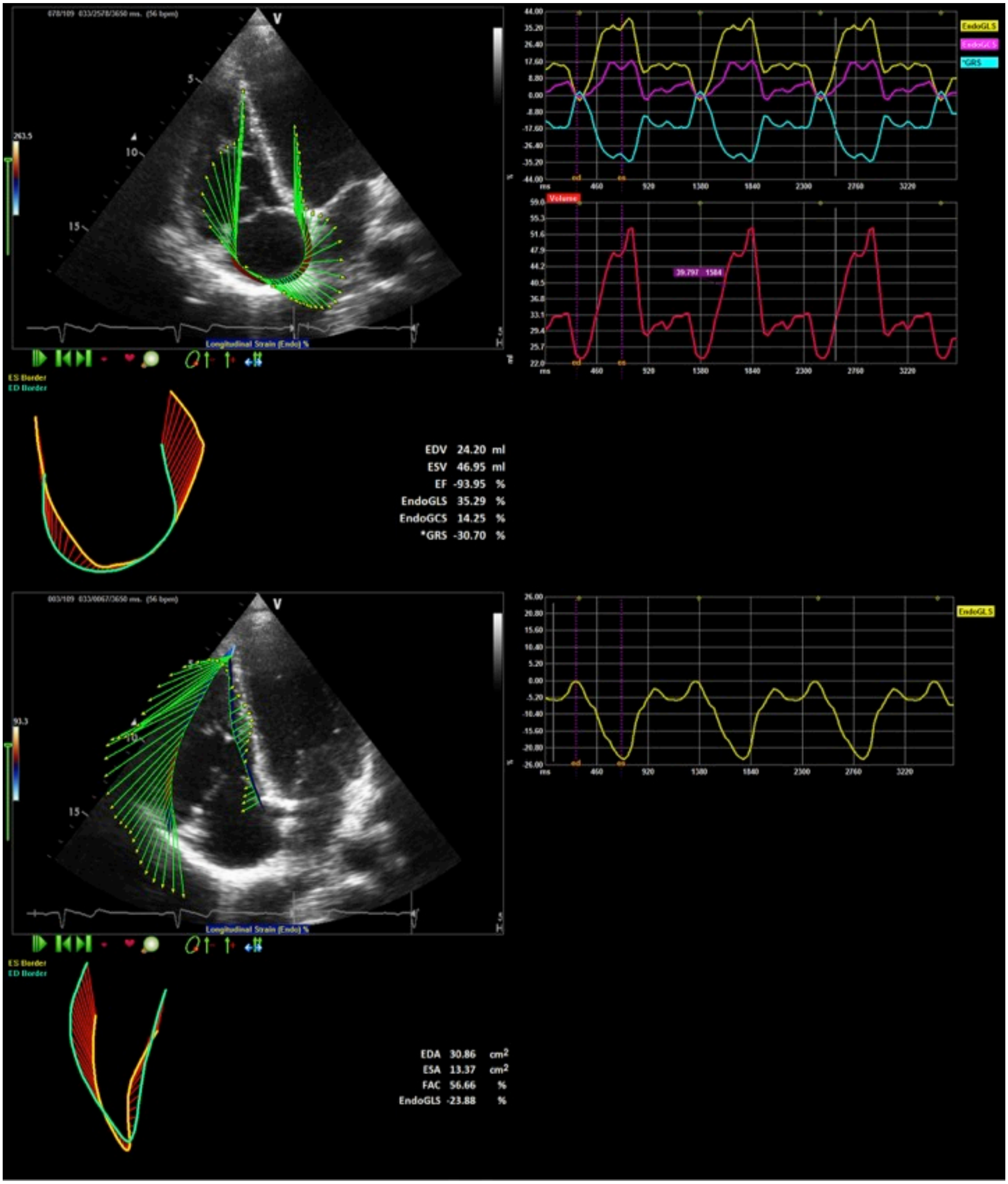

Supplementary figure 1. **Strain analysis of the right ventricle and atrium in the apical four-chamber view.**
